# Coronary Heart Disease (CHD) in Elderly Patients: Which Drug to Choose, Ticagrelor and Clopidogrel? A Systematic Review and Meta-Analysis of Randomized Controlled Trials

**DOI:** 10.3390/jcdd8100123

**Published:** 2021-09-30

**Authors:** Mohammed Ahmed Al-Kaif, Abubakar Sha’aban, Nur Aizati Athirah Daud, Ismaeel Yunusa, Mei Li Ng, Muhamad Ali Sk Abdul Kader, Dzul Azri Mohamed Noor, Baharudin Ibrahim

**Affiliations:** 1 Discipline of Clinical Pharmacy, School of Pharmaceutical Sciences, Universiti Sains Malaysia, Penang 11800, Malaysia; mohammed.alkaif@student.usm.my (M.A.A.-K.); aizati@usm.my (N.A.A.D.); dzulazri@usm.my (D.A.M.N.); 2 Department of Clinical Pharmacy and Pharmacy Practice, Faculty of Pharmaceutical Sciences, Ahmadu Bello University, Zaria 810107, Nigeria; 3 College of Pharmacy, University of South Carolina, Columbia, SC 29208, USA; iyunusa@mailbox.sc.edu; 4 Advanced Medical and Dental Institute, Universiti Sains Malaysia, Penang 13200, Malaysia; nmeili07@nus.edu.sg; 5 Department of Cardiology, Penang General Hospital, Penang 10990, Malaysia; mdali_sheikh@hotmail.com; 6 Faculty of Pharmacy, University of Malaya, Kuala Lumpur 50603, Malaysia

**Keywords:** ticagrelor, clopidogrel, systematic review, meta-analysis, randomized controlled trials

## Abstract

Background: A new generation P2Y12 receptor inhibitor (ticagrelor) is recommended in current therapeutic guidelines to treat patients with coronary heart disease (CHD). However, it is unknown if ticagrelor is more effective than clopidogrel in elderly patients. Therefore, a systematic review was done to assess the effectiveness and safety of ticagrelor and clopidogrel in older patients with CHD to determine the appropriate antiplatelet treatment plan. Methodology: We performed a systematic review of randomized controlled trials (RCTs) to compare the effectiveness and safety of ticagrelor vs. clopidogrel in elderly patients with CHD. We selected eligible RCTs based on specified study criteria following a systematic search of PubMed and Scopus databases from January 2007 to May 2021. Primary efficacy outcomes assessed were major adverse cardiovascular events (MACEs), myocardial infarction (MI), stent thrombosis (ST), and all-cause death. The secondary outcome assessed was major bleeding events. We used RevMan 5.3 software to conduct a random-effects meta-analysis and estimated the pooled incidence and risk ratios (RRs) with 95% confidence intervals (CIs) for ticagrelor and clopidogrel. Results: Data from 6 RCTs comprising 21,827 elderly patients were extracted according to the eligibility criteria. There was no significant difference in the MACE outcome (incidence: 9.23% vs. 10.57%; RR = 0.95, 95% CI = 0.70–1.28, *p* = 0.72), MI (incidence: 5.40% vs. 6.23%; RR = 0.94, 95% CI= 0.69–1.27, *p* = 0.67), ST (incidence: 2.33% vs. 3.17%; RR = 0.61, 95% CI= 0.32–1.17, *p* = 0.13), and all-cause death (4.29% vs. 5.33%; RR = 0.86, 95% CI = 0.65–1.12, *p* = 0.25) for ticagrelor vs. clopidogrel, respectively. In addition, ticagrelor was not associated with a significant increase in the rate of major bleeding (incidence: 9.98% vs. 9.33%: RR = 1.37, 95% CI = 0.97–1.94, *p* = 0.07) vs. clopidogrel. Conclusions: This study did not find evidence that ticagrelor is significantly more effective or safer than clopidogrel in elderly patients with CHD.

## 1. Introduction

The global burden of cardiovascular diseases has increased the global death rate, which has risen from 12.1 million (11.4–12.6 million) in 1990 to 18.6 million (17.1–19.7 million) in 2019 [1]. The incidence of CHD has also increased significantly [1]. CHD is a common condition associated with a high rate of sudden death and serious consequences, with almost 9 million fatalities worldwide in 2017, accounting for roughly 16% of all deaths [2]. Elderly patients account for a large proportion of those affected, far more than the enrolment rate in RCTs [3]. The risk score for recurrent ischemic events shows a three-fold higher event rate for an 80-year-old patient than for a 60-year-old [4]. The risk of bleeding was also increased significantly in elderly patients [5]. CHD also has imposed significant social and economic burdens all around the world. In recent years, the incidence of coronary heart disease has increased, especially in developing regions. Standardized management of CHD is the key to improving prognosis and reducing mortality [6]. Current guidelines for the management of patients with CHD recommend the use of dual antiplatelet therapy (DAPT) to reduce coronary thrombosis and mortality in patients who had acute coronary syndrome (ACS) or who have undergone percutaneous coronary intervention (PCI) [7]. For a long time, DAPT for patients with CHD has generally been aspirin combined with an adenosine diphosphate (ADP) receptor antagonist. Clopidogrel is the first choice for treatment, while ticagrelor is a relatively newer alternative [6]. PLATO trial in adult patients with ACS showed that ticagrelor was more effective than clopidogrel for 12 months [6,8]. Several recent literature reviews emphasized the importance of using ticagrelor as an alternative to clopidogrel to avoid clopidogrel resistance and improve clinical outcomes for some patients [9,10,11]. In addition, anti-platelet drugs increase the risk of bleeding especially in elderly patients [6,12,13]. Therefore, the choice of P2Y12 inhibitors should be carefully considered in this population. A recent randomized controlled trial (RCT) demonstrated the effect of platelet inhibitors in elderly patients with NSTE-ACS and observed no difference in ischemic events between potent P2Y12 inhibitors and clopidogrel, but noted a significant bleeding risk in potent P2Y12 inhibitors [14]. In addition, a recent observational study showed that the use of ticagrelor was associated with an increased risk of death and bleeding among elderly patients with myocardial infarction. Therefore, this study recommended the necessity of conducting a randomized study to examine the effects of ticagrelor on elderly patients [15]. Thus, the effect of ticagrelor vs. clopidogrel on ischemic and bleeding events was verified, but the results were different between studies. Therefore, we conducted a systematic review and meta-analysis to evaluate the outcomes of ticagrelor vs. clopidogrel therapy in elderly patients with CHD to provide more appropriate evidence for antiplatelet therapy in this population.

## 2. Materials and Methods

We conducted a meta-analysis of randomized controlled trials (RCTs) based on the Preferred Reporting Items for Systematic Review and Meta-Analysis (PRISMA) guideline [16]. The protocol was registered in PROSPERO (CRD42021257283).

### 2.1. Search Strategy

Relevant publications were searched from PubMed and Scopus from January 2007 to May 2021. The following keywords were used to find all publications related to this study: “ticagrelor OR AZD6140 and clopidogrel” and (“elderly OR old age patients”) and (“acute coronary syndrome” OR “myocardial infarction” OR “unstable angina” OR “coronary heart disease” OR “coronary artery disease” OR “acute coronary syndrome” OR “myocardial infarction” OR “unstable angina”). Disagreements were settled by negotiating between two reviewers or consulting a third author. No language restrictions were enforced.

### 2.2. Eligibility Criteria

The following inclusion and exclusion criteria were used to evaluate eligible studies.

#### 2.2.1. Inclusion Criteria

The type of studies included in this meta-analysis were RCTs with complete data. The study population was elderly patients (mean age ≥ 60 years) with CHD, including those assigned to either ticagrelor or clopidogrel as secondary prevention after PCI with or without coronary stenting or after ACS admission. During the follow-up period following PCI, they reported clinical outcomes on effectiveness and safety as endpoints. The follow-up period used in the studies was 6–12 months.

#### 2.2.2. Exclusion Criteria

Studies were excluded if they were non-randomized or poorly designed; animal experiments; had incomplete data or errors; repeated publications; studies without clinical outcomes of interest, editorials, reviews, case reports, commentary letters, economic evaluations; and studies that failed to provide the information required for this review.

### 2.3. Outcome Assessment

The primary outcomes are MACE, MI, ST, and all-cause deaths. MI was defined according to the American College of Cardiology Foundation/American Heart Association/European Society of Cardiology/World Heart Federation task force [17]. In addition, MACE includes cardiovascular death, stroke, and MI.

The secondary outcome was major bleeding for safety assessment, and its definition in the studies differ based on the Thrombolysis in Myocardial Infarction (TIMI) major bleeding, Platelet inhibition, and Patient Outcomes (PLATO) major bleeding, bleeding requiring transfusion or prolonging hospitalization, and International Society on Thrombosis and Haemostasis definitions [18]. In addition, for each study, the risk ratio (RR) was abstracted.

### 2.4. Study Selection Process and Data Extraction

We downloaded all references for consolidation, elimination of duplicates, and further analysis. First, title and abstract screening (Level 1) was done by two authors (M.A.A. and A.S.) independently to exclude documents that do not meet the inclusion criteria. Full text of articles that passed the Level 1 screening was then independently screened against the eligibility criteria (Level 2). Before deciding to include them, a third author (B.I.) was consulted to reach a decision and resolve inconsistencies in the literature. At least two reviewers then independently extracted data from eligible studies (Level 3).

### 2.5. Risk of Bias Assessment

The Cochrane Collaboration RoB 2.0 tool was used to assess the quality of all studies [19]. Two authors (M.A.A. and A.S.) independently assessed research quality based on the Cochrane Guide to Systematic Review; judgment, comparability, and outcomes were selected independently using the Cochrane Risk of Bias tool, and any disagreements were resolved unanimously.

### 2.6. Statistical Analysis

RevMan 5.3 statistical software was used for statistical analysis, including the risk ratio (RR) with 95% confidence intervals (CI) [20].

As the studies were conducted in different geographic locations and at other times and populations composed of heterogeneous groups using different criteria for selection, statistical heterogeneity was examined using The Higgins I-square (I^2^) statistic with a *p*-value to assess heterogeneity among studies using a random-effects model [21]. High heterogeneity was considered to be present when the I^2^ index was >75% [21].

The publication bias was visually examined using funnel plots, but formal testing was not feasible due to the small number of studies [22]. Statistical significance for this meta-analysis was set at 0.05.

Sensitivity analysis was performed using the “leave-1-out” method, excluding the studies one by one; for example, the Gimbel et al., (2020) study was excluded, and it is noted if there are any differences obtained in the results [23].

## 3. Results

### 3.1. Study Selection

**Figure 1:**
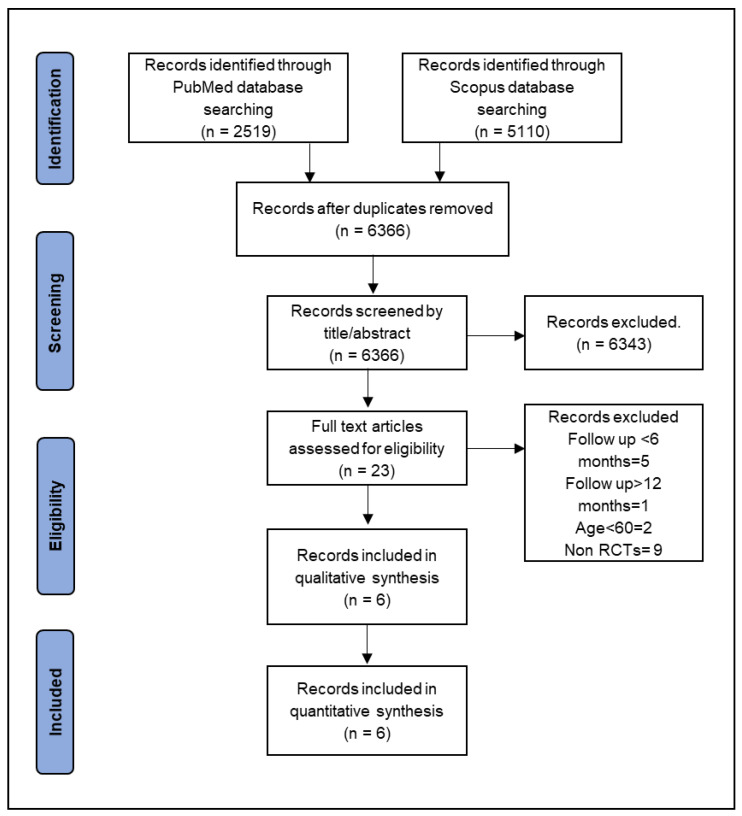
Flow chart of articles screening.

The search primarily identified 6366 articles (after duplicates were removed) that were potentially relevant to the study. With the removal of review articles and after reviewing the title and abstract, 23 articles were assessed. According to the eligibility criteria, a total of 6 articles published between 2009 and 2020 were included in this study (Figure 1).

### 3.2. Characteristics of Studies

**Figure 2:**
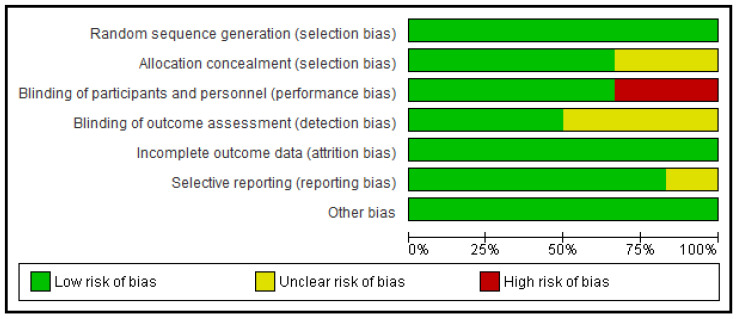
Graph of risk of bias.

The study characteristics of the research publications and samples are included in Table 1. Detailed information about patient characteristics can be found in Table 2. All trials included were generally considered to be of a low bias risk based on the RoB2 tool of the Cochrane (Figure 2). All the six RCTs included [6,14,24,25,26,27] compared two groups, making a total of 10,936 cases in the ticagrelor group and 10,891 cases in the clopidogrel group. The mean age of the patients included in the six studies was 68.6 years old, with a follow-up of 12 months.

### 3.3. Primary Outcomes: MACE, MI, ST and All-Cause Death

**Figure 3:**
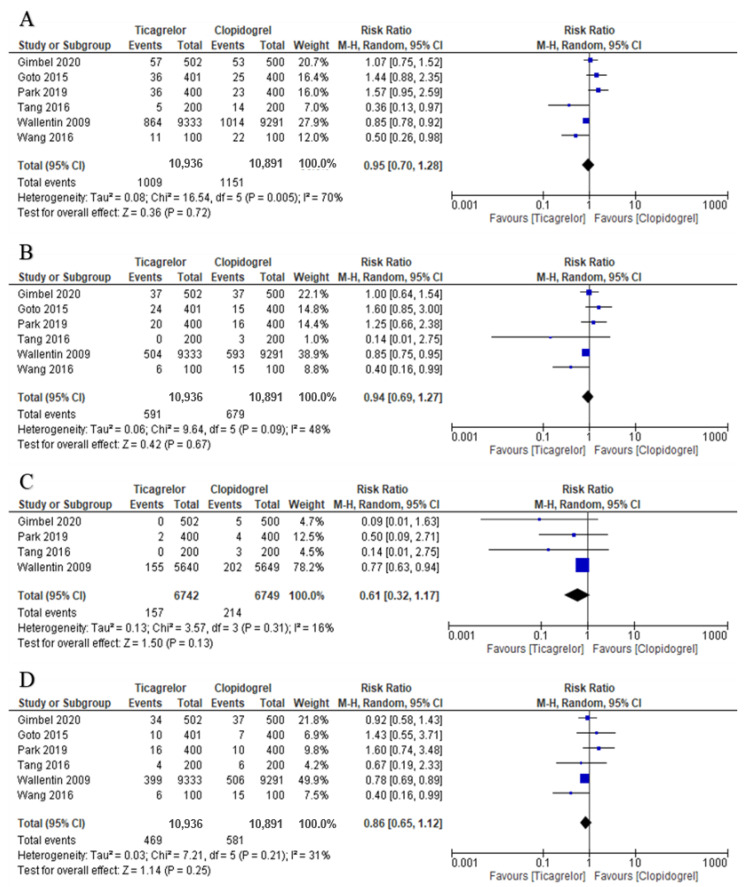
The forest plots for (**A**): MACE; (**B**): MI; (**C**): stent thrombosis; (**D**): all-cause mortality in elderly patients treated with Ticagrelor versus clopidogrel.

Results of this analysis did not show any significant difference in the outcomes between ticagrelor and clopidogrel treatment for MACE (incidence: 9.23% vs. 10.57%; RR = 0.95, 95% CI = 0.70–1.28, *p* = 0.72), MI (incidence: 5.40% vs. 6.23%; RR = 0.94, 95% CI = 0.69–1.27, *p* = 0.67), ST (incidence: 2.33% vs. 3.17%; RR = 0.61, 95% CI = 0.32–1.17, *p* = 0.13), and all-cause death (incidence: 4.29% vs. 5.33%; RR = 0.86, 95% CI = 0.65–1.12, *p* = 0.25) as shown in Figure 3.

### 3.4. Secondary Outcomes: Major Bleeding

**Figure 4:**
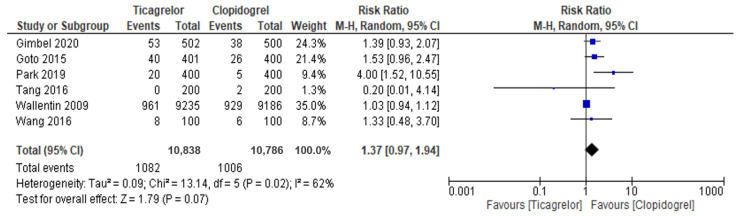
The forest plots for major bleeding in elderly patients treated with Ticagrelor versus clopidogrel.

This meta-analysis did not show any significant difference between ticagrelor and clopidogrel for major bleeding (incidence: 9.98% vs. 9.33%: RR = 1.37, 95% CI = 0.97–1.94, *p* = 0.07) (Figure 4).

### 3.5. Sensitivity Analyses

The sensitivity analysis results showed consistency in some of the studies when excluding studies one by one. Each time a new analysis was performed did not significantly impact the pooled results and conclusions of the study. However, the only difference was that the result representing ST was significant (incidence: 2.52% vs. 3.34%; RR = 0.76, 95% CI = 0.62–0.93, *p* = 0.008) when we excluded the Gimbel et al. study; also, ST was significant (incidence: 0.18% vs. 1.09%; RR = 0.28, 95% CI = 0.07–1.02, *p* = 0.008) when we excluded the Wallentin et al. study. In addition, major bleeding was significant (incidence: 7.55% vs. 4.81%; RR = 1.59, 95% CI = 1.08–2.35, *p* = 0.02) when we excluded the Wallentin et al. study.

## 4. Discussion

We conducted a meta-analysis of RCTs to compare the effectiveness and safety of ticagrelor vs. clopidogrel in elderly patients with CHD. Ticagrelor’s efficacy and safety have been similar to that of clopidogrel. Therefore, our comparison will focus more on the distinctive mechanisms of action of ticagrelor vs. clopidogrel. The mechanism of action of ticagrelor differs from clopidogrel in that clopidogrel acts irreversibly by blocking the P2Y12 receptor [28]. It is absorbed in the intestine, then metabolized by hepatic cytochrome P450 enzymes to active metabolites, and permanently binds to the P2Y12 receptor [29]. Ticagrelor, unlike clopidogrel, does not require activation of the metabolism. Thus, it is less affected by the variability associated with CYP polymorphisms in theory and thus produces a more antiplatelet effect [30,31,32]. Therefore, in elderly patients with low platelet aggregation, coagulation dysfunction, and other high bleeding risks, ticagrelor treatment must be used cautiously [26].

Compared to our findings, the therapeutic benefit of ticagrelor vs. clopidogrel was not significantly different between patients ≥75 years old and those <75 years old, according to a substudy of the PLATO trial. Also, no increase in major bleeding events was observed with ticagrelor vs. clopidogrel in patients aged ≥75 or <75 years (HR 1.02, 95% CI 0.82–1.27) or (HR 1.04, 95% CI 0.94–1.15), respectively [12]. This is similar to our findings, which revealed that ticagrelor was not linked to a significant rate of major bleeding vs. clopidogrel.

In a study comparing ticagrelor and clopidogrel in Asian and non-Asian people with ACS, Kang et al., (2015) found no significant difference in efficacy between the two drugs. [33]. In addition, a recent meta-analysis found no significant difference in effectiveness outcomes between potent P2Y12 (ticagrelor and prasugrel) inhibitors and clopidogrel, even though P2Y12 inhibitors were linked to significant bleeding, posing a risk to older patients [34]. In addition, a meta-analysis of the results of potent P2Y12 inhibitors vs. clopidogrel in elderly ACS patients found that potent P2Y12 inhibitors were more effective in reducing CV death and MI. However, they were not safe and increased bleeding risk compared with clopidogrel [35]. But their study included both ticagrelor and prasugrel, which would not give definitive results that strictly correlate with the use of ticagrelor vs. clopidogrel.

According to one study of elderly ACS patients using DAPT, ischemic events are more common in the acute phase, while bleeding events are more common in the late phase [36]. Therefore, patients are recommended to take aspirin with ticagrelor in the acute phase (during the first months of injury) followed by aspirin and clopidogrel in the late phase. It may be considered an effective and practical strategy to reduce ischemic and bleeding events in elderly patients [37]. This recommendation is supported by several studies where ticagrelor was switched to clopidogrel in ACS patients undergoing PCI [38,39,40]. Still, the effectiveness and safety of this switching require more studies to determine the appropriate time for switching.

## 5. Limitations

The meta-analysis contains some limitations. The hypotheses, inclusion and exclusion criteria, identification of elderly patients, and endpoints of the trials included in our study were slightly different. Furthermore, the dosages of the medications utilized were not considered. Finally, the quality of the literature included was variable. Therefore, this study has limited clinical guidance and is for reference only to clinicians, and its results should be considered exploratory. Further evidence-based studies are needed to verify the effectiveness and safety of ticagrelor vs. clopidogrel in elderly CHD patients.

## 6. Conclusions

This study showed ticagrelor is not significantly more efficacious than clopidogrel in elderly patients with CHD. In addition, it was not associated with significant major bleeding compared to clopidogrel.

## Figures and Tables

**Table 1:** Characteristics of every included study.

Authors	Location	Centres (N.)	Diagnosis	Design of the Study	Age Mean (SD) or Median (IQR)		Follow Up (Months)	Bleeding Classification	Outcome Indication	
					Ticagrelor	Clopidogrel			Ticagrelor	Clopidogrel
Gimbel et al., 2020 [14]	Netherlands	12	NSTE-ACS	RCTs	77.00 (73–82)	77.00 (73–81)	12	PLATO	MACE, MI, Mortality, Bleeding	MACE, MI, ST, Mortality, Bleeding
Goto et al., 2015 [27]	Japan	110	ACS	RCTs	67.00 (12)	66.00 (11)	12	PLATO	MACE, MI, Mortality, Bleeding	MACE, MI, Mortality, Bleeding
Park et al., 2019 [26]	Korea	10	ACS	RCTs	62.50 (11.3)	62.30 (11.5)	12	PLATO	MACE, MI, ST, Mortality, Bleeding	MACE, MI, ST, Mortality, Bleeding
Tang et al., 2016 [25]	China	2	STEMI	RCTs	64.36 (11.4)	64.18 (11.1)	6	TIMI	MACE, Mortality, Bleeding	MACE, MI, ST, Mortality, Bleeding
Wallentin et al., 2009 [6]	USA	862	ACS	RCTs	62.00	62.00	12	PLATO	MACE, MI, ST, Mortality, Bleeding	MACE, MI, ST, Mortality, Bleeding
Wang and Wang 2016 [24]	China	1	ACS	RCTs	79.00 (76–85)	80.00 (74–86)	12	PLATO	MACE, MI, ST, Mortality, Bleeding	MACE, MI, Mortality, Bleeding

T—Ticagrelor; C—Clopidogrel; N.—number; MACE—major adverse cardiac events; MI—Myocardial infarction; ST—stent thrombosis; NSTE-ACS—non-ST-elevation acute coronary syndrome; STEMI—ST-elevation myocardial infraction; RCTs—randomized controlled trials; SD—standard deviation; IQR—interquartile range.

**Table 2:** Baseline characteristics of patients in each trial.

	Gimbel et al., 2020 [14]		Goto et al., 2015 [27]		Park et al., 2019 [26]		Tang et al., 2016 [25]		Wallentin et al., 2009 [6]		Wang and Wang 2016 [24]	
Medication	Ticagrelor	Clopidogrel	Ticagrelor	Clopidogrel	Ticagrelor	Clopidogrel	Ticagrelor	Clopidogrel	Ticagrelor	Clopidogrel	Ticagrelor	Clopidogrel
Loading dose (mg)	180	300,600	180	300	180	600	180	600	180	300	180	300
Maintenance dose (mg)	90	75	90	75	90	75	90	75	90	75	90	75
Patient number	502	500	401	400	400	400	200	200	9333	9291	100	100
Male (%)	325 (65)	313 (63)	ND	ND	297 (74.2)	302 (75.5)	142 (71)	146 (73)	ND	ND	ND	ND
Female (%)	177 (35)	187 (37)	95 (23.7)	93 (23.3)	ND	ND	ND	ND	2655 (28.4)	2633 (28.3)	31 (31)	34 (34)
BMI (Kg/m^2^)	26·9 (4·2)	26·7 (4·0)	23.7 (15.6–43.4)c	23.6 (14.2–38.6)	24.6 ± 3.0	24.9 ± 3.2	ND	ND	27 (13–68)	27 (13–70)	ND	ND
Body weight (kg)	ND	ND	63 (35–104)	62 (36–109)	ND	ND	ND	ND	80.0 (28–174)	80.0 (29–180)	ND	ND
Weight < 60 kg (%)	30 (6)	35 (7)	154 (38.4)	152 (38.0)	ND	ND	ND	ND	652 (7.0)	660 (7.1)	ND	ND
Risk factors												
Diabetes mellitus (%)	150 (30)	146 (29)	154 (38.4)	124 (31.0)	116 (29.0)	100 (25.0)	58 (29)	42 (21)	2326 (24.9)	2336 (25.1)	42 (42)	39 (39)
Dyslipidemia (%)	325 (65)	323 (65)	314 (78.3)	289 (72.3)	208 (52.0)	194 (48.5)	88 (44)	74 (37)	4347 (46.6)	4342 (46.7)	84 (84)	79 (79)
Hypertension (%)	365 (73)	362 (73)	305 (76.1)	290 (72.5)	223 (55.8)	193 (48.2)	122 (61)	116 (58)	6139 (65.8)	6044 (65.1)	79 (79)	82 (82)
Smoking (%)	62 (13)	67 (14)	151 (37.7)	157 (39.3)	146 (36.5)	139 (34.8)	116 (58)	124 (62)	3360 (36.0)	3318 (35.7)	37 (37)	41 (41)
**Previous medical history**												
PCI (%)	122 (24)	98 (20)	45 (11.2)	42 (10.5)	41 (10.2)	31 (7.8)	ND	ND	1272 (13.6)	1220 (13.1)	3 (3)	6 (65)
MI (%)	136 (27)	121 (24)	33 (8.2)	31 (7.8)	25 (6.2)	20 (5.0)	16 (8)	10 (5)	1900 (20.4)	1924 (20.7)	17 (17)	15 (15)
Peripheral arterial disease (%)	49 (10)	62 (12)	13 (3.2)	14 (3.5)	4 (1.0)	2 (0.5)	10 (5)	8 (4)	566 (6.1)	578 (6.2)	5 (5)	7 (7)
Congestive heart failure (%)	ND	ND	30 (7.5)	28 (7.0)	10 (2.5)	6 (1.5)	ND	ND	513 (5.5)	537 (5.8)	13 (13)	9 (9)
Angina pectoris (%)	ND	ND	102 (25.4)	110 (27.5)	ND	ND	114 (57)	104 (52)	ND	ND	40 (40)	36 (36)
Atrial fibrillation/flutter (%)	ND	ND	ND	ND	ND	ND	18 (9)	22 (11)	ND	ND	ND	ND
Non-hemorrhagic stroke (%)	ND	ND	27 (6.7)	28 (7.0)	ND	ND	ND	ND	353 (3.8)	369 (4.0)	11 (11)	10 (10)
Peptic ulcer disease (%)	ND	ND	37 (9.2)	37 (9.3)	ND	ND	ND	ND	ND	ND	ND	ND
Gastrointestinal bleeding (%)	ND	ND	6 (1.5)	7 (1.8)	1 (0.2)	0 (0.0)	ND	ND	ND	ND	ND	ND
Asthma (%)	ND	ND	12 (3.0)	14 (3.5)	12 (3.0)	3 (0.8)	ND	ND	267 (2.9)	265 (2.9)	ND	ND
Dyspnea (%)	ND	ND	32 (8.0)	41 (10.3)	ND	ND	ND	ND	1412 (15.1)	1358 (14.6)	ND	ND
CABG (%)	86 (17)	84 (17)	5 (1.2)	1 (0.3)	4 (1.0)	3 (0.8)	ND	ND	532 (5.7)	574 (6.2)	0 (0)	0 (0)
Ischaemic stroke (%)	25 (5)	22 (4)	ND	ND	24 (6.0)	16 (4.0)	ND	ND	ND	ND	ND	ND
Transient ischaemic attack (%)	38 (8)	37 (7)	ND	ND	ND	ND	32 (16)	34 (17)	ND	ND	16 (16)	14 (14)
Chronic renal disease (%)	ND	ND	18 (4.5)	20 (5.0)	6 (1.5)	1 (0.2)	ND	ND	379 (4.1)	406 (4.4)	ND	ND
COPD (%)	49 (10)	61 (12)	7 (1.7)	10 (2.5)	ND	ND	ND	ND	555 (5.9)	530 (5.7)	ND	ND
Gout (%)	ND	ND	23 (5.7)	23 (5.7)	5 (1.2)	4 (1.0)	ND	ND	272 (2.9)	262 (2.8)	ND	ND
**Diagnosis**												
NSTEMI (%)	424 (86)	423 (86)	66 (16.5)	74 (18.5)	148 (37.0)	155 (38.8)	ND	ND	4005 (42.9)	3950 (42.5)	44 (44)	47 (47)
STEMI (%)	ND	ND	205 (51.1)	210 (52.5)	170 (42.5)	156 (39.0)	ND	ND	3496 (37.5)	3530 (38.0)	37 (37)	32 (32)
Unstable angina (%)	52 (11)	54 (11)	119 (29.7)	109 (27.3)	82 (20.5)	89 (22.2)	ND	ND	1549 (16.6)	1563 (16.8)	19 (19)	21 (21)
Positive troponin (%)	ND	ND	309 (77.1)	298 (74.5)	338 (84.5)	333 (83.3)	ND	ND	7965/9333 (85.3)	7999/9291 (86.1)	ND	ND
**ECG findings**												
Persistent ST-segment elevation (%)	ND	ND	218 (54.4)	225 (56.3)	ND	ND	ND	ND	3497 (37.5)	3511 (37.8)	ND	ND
ST-segment depression (%)	ND	ND	188 (46.9)	153 (38.3)	ND	ND	ND	ND	4730 (50.7)	4756 (51.2)	ND	ND
T-wave inversion (%)	ND	ND	142 (35.4)	126 (31.5)	ND	ND	ND	ND	2970 (31.8)	2975 (32.0)	ND	ND
**Discharge medications**												
Organic nitrate (%)	ND	ND	344 (85.8)	353 (88.3)	ND	ND	ND	ND	7181 (76.9)	7088 (76.3)	ND	ND
Vitamin K antagonist (%)	62 (12)	65 (13)	ND	ND	ND	ND	ND	ND	ND	ND	ND	ND
Proton pump inhibitor (%)	446 (91)	446 (90)	167 (41.6)	175 (43.8)	12 (3.0)	8 (2.0)	ND	ND	4233 (45.4)	4128 (44.4)	31 (31)	33 (33)
β-blocker (%)	ND	ND	40 (10)	44 (11.1)	275 (68.8)	297 (74.2)	82	96	8339 (89.3)	8336 (89.7)	69 (69)	74 (74)
ACE inhibitor (%)	ND	ND	67 (16.7)	64 (16.0)	ND	ND	ND	ND	7090 (76.0)	6986 (75.2)	61 (61)	67 (67)
Angiotensin receptor blocker (%)	ND	ND	102 (25.4)	95 (23.8)	163 (40.8)	171 (42.8)	ND	ND	1143 (12.2)	1125 (12.1)		
Statin (%)	ND	ND	215 (53.6)	205 (51.3)	354 (88.5)	369 (92.2)	198	199	8373 (89.7)	8289 (89.2)	83 (83)	79 (79)
Calcium channel blocker (%)	ND	ND	117 (29.2)	109 (27.3)	90 (22.5)	90 (22.5)	ND	ND	2769 (29.7)	2789 (30.0)	69 (69)	63 (63)
**Procedure**												
Coronary angiography (%)	452 (90)	439 (88	385 (96.0)	378 (95.4)	ND	ND	ND	ND	7599 (81.4)	7571 (81.5)	86 (86)	83 (83)
PCI (%)	242 (48)	232 (46)	340 (84.8)	338 (84.5)	ND	ND	ND	ND	5978 (64.1)	5999 (64.6)	75 (75)	71 (71)
BMS (%)	6 (3)	2 (1)	ND	ND	ND	ND	ND	ND	3921 (42.0)	3892 (41.9)	ND	ND
DES (%)	224 (93)	219 (94)	ND	ND	ND	ND	ND	ND	1719 (18.4)	1757 (18.9)	ND	ND
CABG (%)	87 (17)	78 (16)	9 (2.2)	3 (0.8)	ND	ND	ND	ND	931 (10.0)	968 (10.4)	0 (0)	0 (0)

T—ticagrelor; C—clopidogrel; PCI—percutaneous coronary intervention; MI—myocardial infraction; CABG—coronary artery bypass grafting; COPD—Chronic obstructive pulmonary disease; NSTEMI—non-ST-elevation myocardial infraction; STEMI—ST-elevation myocardial infraction; BMS— bare metal stent; DES— drug eluting stent; ND—no data.
